# Drugs acting on the renin–angiotensin–aldosterone system (RAAS) and deaths of COVID-19 patients: a systematic review and meta-analysis of observational studies

**DOI:** 10.1186/s43044-022-00303-8

**Published:** 2022-09-06

**Authors:** Ruchika Sharma, Anoop Kumar, Jaseela Majeed, Ajit K. Thakur, Geeta Aggarwal

**Affiliations:** 1 Centre for Precision Medicine and Pharmacy, Delhi Pharmaceutical Sciences and Research University, New Delhi, 110017 India; 2 Department of Pharmacology, Delhi Pharmaceutical Sciences and Research University (DPSRU), New Delhi, 110017 India; 3 Department of Pharmaceutical Management, Delhi Pharmaceutical Sciences and Research University, New Delhi, 110017 India; 4 Department of Pharmaceutics, Delhi Pharmaceutical Sciences and Research University (DPSRU), New Delhi, 110017 India

**Keywords:** COVID-19, Angiotensin-converting enzyme inhibitor (ACEi), Angiotensin receptor blocker (ARB), Mortality

## Abstract

**Background:**

Angiotensin-converting enzyme inhibitors (ACEi) and angiotensin receptor blockers (ARBs) are two of the most commonly used antihypertensive drugs acting on the renin–angiotensin–aldosterone system (RAAS). Previous research has shown that RAAS inhibitors increase the expression of angiotensin-converting enzyme, a cellular receptor for the severe acute respiratory syndrome coronavirus 2, raising concerns that the use of ACEi and ARBs in hypertensive patients may increase COVID-19 patient mortality. Therefore, the main aim of the current study was to find out the role of drugs acting on RAAS, particularly ACEi/ARBs in the deaths of COVID-19 patients.

**Results:**

In total, 68 studies were found to be appropriate, reporting a total of 128,078 subjects. The odds ratio was found to be 1.14 [0.95, 1.36], which indicates the non-significant association of ACEi/ARBs with mortality of COVID-19 patients. Further, the association of individual ACEi/ARBs with mortality of COVID-19 patients was also found non-significant. The sensitivity analysis results have shown no significant effect of outliers on the outcome.

**Conclusions:**

Based on available evidence, ACEi/ARB were not significantly associated with deaths of COVID-19 patients.

**Supplementary Information:**

The online version contains supplementary material available at 10.1186/s43044-022-00303-8.

## Key points

- The role of drugs that are used in the management of hypertension and cardiovascular diseases in the deaths of COVID-19 patients is unclear so far
- The results of the current analysis have found the non-significant role of these drugs in the deaths of COVID-19 patients
- Based on current evidence, we suggest to continue the use of drugs particularly ACEi/ARB in the management of hypertension and cardiovascular diseases in COVID-19 patients

## Background

Recent, highly contagious novel coronavirus (2019-nCoV) caused by SARS-CoV-2 emerged from Wuhan, China, and rapidly spread over more than 100 countries has caused unprecedented health concerns all over the globe. The first case was recorded in November 2019, and the World Health Organization (WHO) declared a pandemic and a global public health emergency on March 11, 2020 [1]. The virus spread continuously despite many drastic containment measures (complete lockdown, curfews, etc.). On March 13, 2022, more than 456,797,217 cases of COVID-19 were reported across the globe, resulting in approximately 6,043,094 deaths [2]. Health authorities all over the world are struggling to develop possible prevention and therapeutic measures [2, 3]. Fortunately, vaccines are developed and used as a preventive measure across the globe. However, still there is no specific drug available for the treatment of infected patients with SARS-CoV-2. There are a number of research questions that are unanswered so far related to this infection. It has been observed that COVID-19 patients with co-morbid conditions such as diabetes (DM), hypertension (HT), or cardiovascular disease (CVD) are more prone to death [3, 4]. There are a number of explanations for this. It has also been hypothesized that the use of medicines in the management of co-morbid conditions also could be one of the reasons. Hypertension and cardiovascular diseases are the most common co-morbid conditions, and the most commonly used drugs in the management of these conditions are acting on the renin–angiotensin–aldosterone system (RAAS) such as ACEi/ARBs. SARS-CoV-2 enters the cell through the host's angiotensin-converting enzyme (ACE) [4], and drugs acting on the RAAS (ACEi and ARB) may boost ACE2 expression, resulting in greater SARS-CoV-2 binding [5]. The enhanced binding of SARS-CoV-2 to the host could result in severe symptoms or even deaths of COVID-19 patients. The pieces of evidence have been primarily contentious up to this point. Some studies have also shown the protective effect of these medicines in COVID-19 patients [4, 6], whereas some studies have concluded a higher mortality rate [7–11].

To the best of our knowledge, few meta-analyses have also been conducted to find out the association of ACEi/ARBs in the deaths of COVID-19 patients. However, a number of included studies are too less to draw any valid conclusion. Further, some meta-analyses have also included different designs of studies. Therefore, we have performed a systematic review and meta-analysis of observational studies to find out the exact association of ACEi/ARB in the deaths of COVID-19 patients.

## Methods

### Search strategy

PubMed, Google Scholar, and MedRxiv preprint server were used to identify relevant studies from December 2019 to January 2022 with proper MeSH terms. The MeSH phrases or keywords with Boolean operators were used as followings “(COVID19)” OR “(COVID-19)” OR “(COVID-19 VIRUS INFECTION)” OR “(COVID19 INFECTION)” OR “(SARS COVID 19 INFECTION)” OR “(2019 NOVEL CORONAVIRUS INFECTION)” OR “(SARS COVID DISEASE)” OR “(COVID-19 DISEASE)” AND “(ACE)” OR “(ARB)” OR “(ANGIOTENSIN CONVERTING ENZYME)” OR “(ANGIOTENSIN RECEPTOR BLOCKERS)” which were used are presented in Additional file 1: Table S1. This study was carried out according to the PRISMA [12] and STROBE guidelines [13].

### Eligibility criteria

The studies were included or excluded as per the defined inclusion and exclusion criteria. The inclusion criteria include COVID-19 patients, age above 18 years, use of ACEi/ARB classes of drugs alone or in combinations, one of the outcomes was death. The studies were excluded if published other than in the English language, review articles, meta-analyses, case reports, letters, comments or opinions, animal studies, death was not reported as one of the outcomes and editorials.

### Screening

The screening of relevant studies as per inclusion and exclusion criteria was done independently by two authors (RS and AK). The PRISMA guideline was followed, and a selection of studies based on titles, abstracts, and full texts was presented in the PRISMA flow chart. The conflict among the authors was resolved after discussion with third (JM), fourth (AKT), and fifth authors (GA).

### Quality assessment

The Newcastle–Ottawa (Questionnaire) Scale (NOS) was used to determine the quality of the studies and measuring the risk of bias in cohort and case–control studies [14]. Three reviewers (RS, AK, and JM) have conducted quality assessments of included studies, and disagreements were resolved after discussion with the fourth (AKT) and fifth (GA) authors. The following are the major components used for the quality assessment: comparability, selection of non-exposed cohort, representativeness of the exposed cohort, ascertainment of exposure, outcome assessment, demonstration that the outcome of interest was not present at the start of the study, adequacy of cohort follow-up and follow-up time. The quality rating scale runs from 0 to 10, with a score of > 7 stars indicating high-quality content.

### Data extraction

The data were extracted from 68 studies and cross-checked by both authors (RS and AK). The data were extracted in an M.S Excel sheet which contains the columns like authors’ first names, type of study, location of study, total sample size, number of males/females, age groups, the total number of patients in the ACEi/ARBs and Non-ACEi/ARB’s groups, number of subjects died in the ACEi/ARBs and non-ACEi/ARB’s groups.

### Data analysis

The overall estimate was calculated in terms of odds ratios with a 95% confidence interval. The random-effect model was preferred over the fixed-effect model due to variations among included studies. The Chi-square statistic and the I2 z test were used to measure heterogeneity. The funnel plot was used to determine whether there was any publishing bias. The sensitivity analysis was performed to check the effects of outliers on the outcome. For the data analysis, RevMan 5 was employed.

## Results

### Search results and study characteristics

**Fig. 1:**
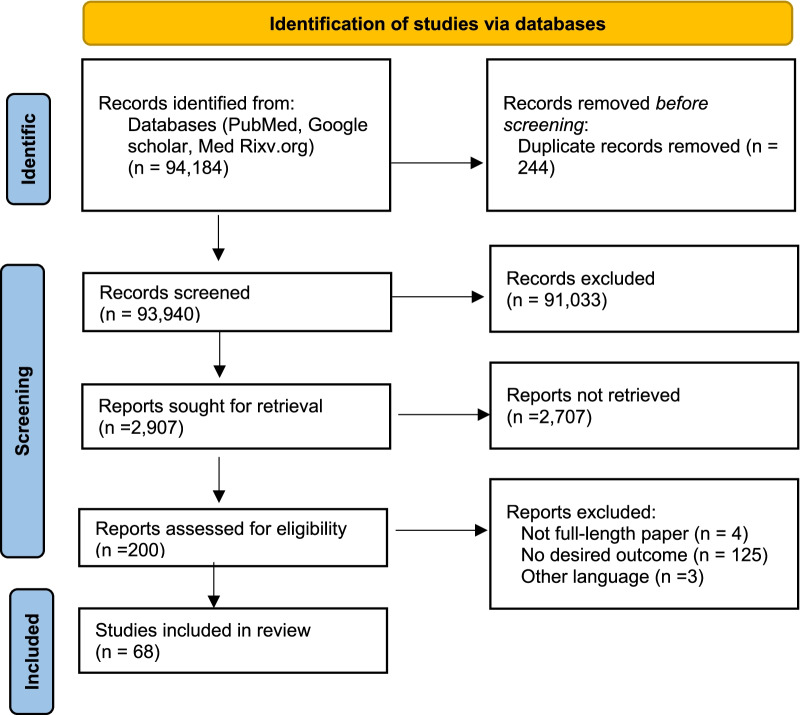
Selection of studies as per the PRISMA Checklist

The initial search identified 94,184 studies. A total of 224 duplicates were found and the remaining 93,940 studies were further screened based on the titles. A total of 2907 studies were found relevant which were further screened based on abstracts. Further, a full text of 200 studies was downloaded, and finally, 68 studies were found relevant for quantitative analysis as per the aim and objective of the current study. Out of these 68 studies, 61 studies were published in peer-reviewed journals, whereas the remaining 7 studies were preprints. The step-by-step screening and selection of studies are presented in Fig. 1 as per the PRISMA flow chart. Out of 68 selected studies [7–11, 15–77], 56 were cohort and the remaining 12 were case–control studies. A total of 128,078 patients were found. The study characteristics of included studies are compiled in Table 1.

**Table 1:** Characteristics of included Studies

S. no.	Reference name	Type of study	Place	Publication status	Male (%)	Female (%)	Total	Median age	(Non-ACE/ARB)	Outcome	(ACE/ARB)	Outcome	References
1	Derington 2021	Cohort	USA	Peer-reviewed	93	7	4969	66	2487	65	2482	61	[15]
2	Ayed 2021	Cohort	Kuwait	Peer-reviewed	85.5	14.5	103	53	93	10	10	5	[16]
3	Senkal 2020	Cohort	Turkey	Peer-reviewed	59.4	40.6	611	57	53	5	105	7	[17]
4	Bae 2020	Cohort	UK	Peer-reviewed	48.8	51.2	347	52	230	12	117	17	[18]
5	Baker 2021	Cohort	UK	Peer-reviewed	54	46	311	75	233	63	78	17	[19]
6	Banerjee 2020	Cohort	UK	Peer-reviewed	57.5	42.5	7	61.5	6	0	1	1	[20]
7	Bauer 2021	Case–Control Study	USA	Peer-reviewed	37	63	1449	57.4	1219	198	230	77	[21]
8	Bean 2020	Cohort	UK	Peer-reviewed	57.2	42.8	1200	69.2	801	182	399	106	[22]
9	Braude 2020	Multicenter Observational Study	UK, Italy	Peer-reviewed	59.1	40.9	1371	74	979	257	392	106	[23]
10	Cannata 2020	Observational Retrospective	Italy	Peer-reviewed	NA	NA	280	75	224	39	56	7	[24]
11	Cariou 2020	Cohort	France	Peer-reviewed	64.9	35.1	1317	69.8	580	48	737	92	[25]
12	Cetinkal 2020	Retrospective, Single Center	Turkey	Peer-reviewed	50.43	49.57	341	68.7	140	20	201	29	[26]
13	Chaudhri 2020	Cohort	USA	Peer-reviewed	66	34	300	62	220	25	80	5	[27]
14	Chen C 2020	Cohort	China	Peer-reviewed	47.7	52.3	1182	63	827	95	355	12	[28]
15	Chen M 2020	Case–Control Study	China	Preprint	49.6	50.4	123	28.8	112	28	11	3	[29]
16	Chen Y 2020	Cohort	China	Peer-reviewed	67	33	71	67.25	39	10	32	4	[30]
17	Choi 2020	Case–Control Study	South Korea	Preprint	42.8	57.2	1517	66.5	625	69	892	42	[31]
18	Christiansen 2021	Observational Retrospective	Denmark	Peer-reviewed	42.7	57.3	2802	47	1336	144	1466	138	[32]
19	Conversano 2020	Cohort	Italy	Peer-reviewed	68.6	31.4	191	65	122	101	69	48	[33]
20	Covino 2020	Observational Retrospective	Australia	Peer-reviewed	65.7	34.3	166	74	55	22	111	58	[34]
21	Desai 2021	Observational Retrospective	Italy	Peer-reviewed	66.1	33.9	575	64.8	421	72	154	49	[35]
22	Felice 2020	Case–Control Study	Italy	Peer-reviewed	64.6	35.4	133	72	51	18	82	15	[7]
23	Fosbol 2020	Cohort	Denmark	Peer-reviewed	48	52	4480	62	3585	297	895	181	[8]
24	Genet 2020	Observational Retrospective	USA	Peer-reviewed	63.81	36.19	201	86.3	138	52	63	14	[36]
25	Giacomelli 2020	Prospective, Single Center	Italy	Peer-reviewed	69.1	30.9	233	61	202	34	31	14	[37]
26	Giorgi 2020	Cohort	Italy	Peer-reviewed	50.1	49.9	2653	31.6	1835	109	818	108	[38]
27	Guo 2020	Case–Control Study	China	Peer-reviewed	54	46	187	61.7	168	36	19	7	[9]
28	Hakeam 2021	Cohort	Saudi Arabia	Peer-reviewed	59.5	40.5	102	60.8	33	7	69	15	[39]
29	Hu 2020	Cohort	China	Peer-reviewed		NA	149	56	84	0	65	1	[10]
30	Huang 2020	Cohort	China	Peer-reviewed	45	55	50	60.18	30	3	20	0	[40]
31	Ip 2020	Case–Control Study	USA	Preprint	NA	NA	1129	NA	669	262	460	137	[41]
32	Jung C 2021	Cohort	38 Countries	Peer-reviewed	69	31	324	75	167	85	157	62	[42]
33	Jung S 2020	Cohort	South Korea	Peer-reviewed	44	56	1954	44.6	1577	51	377	33	[43]
34	Khan 2020	Observational Retrospective, Multicenter	Scotland	Peer-reviewed	56.8	43.2	88	72	61	14	27	5	[44]
35	Khera 2020	Observational Retrospective	USA	Preprint	54	46	7933	NA	3346	466	4587	664	[45]
36	Kim 2021	Observational Retrospective	South Korea	Peer-reviewed	53	47	1236	62	608	28	628	23	[46]
37	Lafaurie 2021	Observational Retrospective	UK	Peer-reviewed	22.44	77.56	109	74	36	6	73	9	[11]
38	Lam 2020	Observational Retrospective	USA	Peer-reviewed	90.11	9.89	614	70.5	279	62	335	58	[47]
39	Lee 2020	Cohort	South Korea	Preprint	22.44	77.56	8266	44.36	7289	62	977	50	[48]
40	Li 2020	Case–Control Study	China	Peer-reviewed	52.2	47.80	362	66	247	56	115	21	[49]
41	Liabeuf 2021	Prospective, Single Center	France	Peer-reviewed	58	42	268	73	172	30	96	17	[50]
42	Lim 2020	Cohort	South Korea	Peer-reviewed	70	30	130	67	100	22	30	14	[51]
43	Lopez-Otero 2021	Cohort	Spain	Peer-reviewed	42	58	965	64	755	27	210	11	[52]
44	Matsuzawa 2020	Observational Retrospective	Japan	Peer-reviewed	59.6	40.4	39	60	18	0	21	2	[53]
45	Mehta 2020	Observational Retrospective	USA	Peer-reviewed	50.1	49.9	1705	58.4	1494	34	211	8	[54]
46	Meng 2020	Case–Control Study	China	Peer-reviewed	57.2	42.8	42	66.4	25	1	17	0	[55]
47	Negreira-Caamaao 2020	Observational Retrospective	Spain	Peer-reviewed	51.9	48.1	545	76.5	153	63	392	119	[56]
48	Oussalah 2020	Cohort	France	Peer-reviewed	61	39	147	65	104	9	43	10	[57]
49	Pan 2020	Observational Retrospective	China	Peer-reviewed	50.7	49.3	282	69	241	5	41	4	[58]
50	Rentsch 2020	Cohort	USA	Preprint	54	46	579	66	324	6	255	11	[59]
51	Rezel-Potts 2021	Observational Retrospective	UK	Peer-reviewed	40	60	16,866	62	14,154	667	2712	254	[60]
52	Richardson 2020	Case–Control Study	USA	Peer-reviewed	60.3	39.7	1366	63	953	254	413	130	[61]
53	Rodilla 2020	Observational Retrospective	Spain	Peer-reviewed	57.4	42.6	12,226	67.5	7988	1452	4238	1180	[62]
54	Rosenthal 2020	Cohort	USA	Peer-reviewed	50	50	37,707	57	34,545	7010	3162	345	[63]
55	Sardu 2020	Observational Retrospective	Italy	Peer-reviewed	66.1	33.9	62	58	17	2	45	7	[64]
56	Selã§Uk 2020	Observational Retrospective	Turkey	Peer-reviewed	48.6	51.4	113	67	39	4	74	31	[65]
57	Shah 2020	Retrospective, Single Center	USA	Peer-reviewed	42	58	531	64	324	48	207	38	[66]
58	Soleimani 2020	Cohort	USA	Peer-reviewed	83.5	16.5	254	66.4	132	35	122	33	[67]
59	Son 2020	Observational Retrospective	Korea	Peer-reviewed	50.8	49.2	102	64	25	8	77	30	[68]
60	Tan 2020	Case–Control Study	China	Peer-reviewed	51	49	100	67	69	11	31	0	[69]
61	Wang 2020	Case–Control Study	China	Peer-reviewed	52	48	210	64	129	5	81	7	[70]
62	Xu 2020	Observational Retrospective	China	Peer-reviewed	53	47	101	65	61	21	40	11	[71]
63	Yang 2020	Observational Retrospective	China	Peer-reviewed	49	51	251	66	208	19	43	2	[72]
64	Yuan 2020	Observational Retrospective	China	Peer-reviewed	NA	NA	260	NA	130	22	130	6	[73]
65	Zeng 2020	Case–Control Study	China	Preprint	49.4	50.6	274	66.57	246	19	28	2	[74]
66	Zhang 2020	Retrospective, Multi-Center Study	China	Peer-reviewed	54	46	1128	64	940	92	188	7	[75]
67	Zhong 2020	Cohort	China	Peer-reviewed	44.5	55.5	126	66.3	89	15	37	6	[76]
68	Zhou 2020	Cohort	China	Peer-reviewed	53	47	2718	64.8	1812	272	906	70	[77]

### Quality evaluation

The Newcastle–Ottawa Scale was used to determine the quality of the studies. The study's cohort and case–control classes were assessed on a scale of 0 to 10, with low risk of bias (8–10), moderate risk (5–7), and high risk (0–4) assigned to each. A total of 52 studies were found to be of excellent quality, and 16 studies were of fair quality as compiled in Table 2. In the case of cohort studies, 49 studies were found to be excellent and 7 were of fair quality, whereas in the case of case–control studies, out of 12 studies, 3 studies were found to be excellent and the remaining 9 were of fair quality.

**Table 2:** Quality assessment using the Newcastle–Ottawa scale

S. no.	References	Selection				Compatibility	Outcome			Total score	Quality of the study	References
		Representativeness of the exposed cohort	Selection of the non-exposed cohort	Ascertainment of the exposure	Outcome status at start of study		Assessment of the outcome	Length of follow-up	Adequacy of follow-up			
*Cohort study*												
1	Derington 2021	*	*	*	*	*	*	*	*	9	Excellent	[15]
2	Ayed 2021	*	*	*	*	*	*	*	*	8	Excellent	[16]
3	Åženkal 2020	*	*	*	*	*	*	*	*	8	Excellent	[17]
4	Bae 2020	*	*	*	*	**	*	*	*	9	Excellent	[18]
5	Baker 2021	*	*	*	*	**–**	*	*	*	7	Fair	[19]
6	Banerjee 2020	*	*	*	*	*	*	*	*	8	Excellent	[20]
7	Bean 2020	*	*	*	*	*	*	*	*	8	Excellent	[22]
8	Braude 2020	*	*	*	*	*	*	*	*	8	Excellent	[23]
9	Cannata 2020	*	*	*	*	*	*	*	*	8	Excellent	[24]
10	Cariou 2020	*	*	*	*	*	*	*	*	8	Excellent	[25]
11	Cetinkal 2020	*	*	*	*	**	*	*	*	9	Excellent	[26]
12	Chaudhri 2020	*	*	*	*	*	*	*	*	8	Excellent	[27]
13	Chen C 2020	*	*	*	*	*	*	*	*	8	Excellent	[28]
14	Chen Y 2020	*	*	*	*	*	*	*	*	8	Excellent	[30]
15	Christiansen 2021	*	*	*	*	*	*	*	*	8	Excellent	[32]
16	Conversano 2020	*	*	*	*	**	*	*	*	9	Excellent	[33]
17	Covino 2020	*	*	*	*	*	*	*	*	8	Excellent	[34]
18	Desai 2021	*	*	*	*	*	*	*	*	8	Excellent	[35]
19	Fosbol¸ 2020	*	*	*	*	**	*	*	**–**	8	Excellent	[8]
20	Genet 2020	*	*	*	*	**–**	*	*	*	7	Fair	[36]
21	Giacomelli 2020	*	*	*	*	*	*	*	*	8	Excellent	[37]
22	Giorgi 2020	*	*	*	*	*	*	*	*	8	Excellent	[38]
23	Hakeam 2021	*	*	*	*	**	*	*	*	9	Excellent	[39]
24	Hu 2020	*	*	*	*	*	*	*	*	8	Excellent	[10]
25	Huang 2020	*	*	*	*	**–**	*	*	*	7	Fair	[40]
26	Jung C 2021	*	*	*	*	**	*	*	*	9	Excellent	[42]
27	Jung S 2020	*	*	*	*	**	*	*	*	9	Excellent	[43]
28	Khan 2020	*	*	*	*	*	*	*	*	8	Excellent	[44]
29	Khera 2020	*	*	*	*	*	*	*	*	8	Excellent	[45]
30	Kim 2021	*	*	*	*	*	*	*	*	8	Excellent	[46]
31	Lafaurie 2021	*	*	*	*	**	*	*	*	9	Excellent	[11]
32	Lam 2020	*	*	*	*	*	*	*	*	8	Excellent	[47]
33	López-Otero 2021	*	*	*	*	*	*	*	*	8	Excellent	[52]
34	Lee 2020	*	*	*	*	**	*	*	*	9	Excellent	[48]
35	Liabeuf 2021	*	*	*	*	*	*	*	*	8	Excellent	[50]
36	Lim 2020	*	*	*	*	*	*	*	*	8	Excellent	[51]
37	Matsuzawa 2020	*	*	*	*	*	*	*	*	8	Excellent	[53]
38	Mehta 2020	*	*	*	*	**–**	*	*	*	7	Fair	[54]
39	Negreira-Caamaao 2020	*	*	*	*	*	*	*	*	8	Excellent	[56]
40	Oussalah 2020	*	*	*	*	**	*	*	*	9	Excellent	[57]
41	Pan 2020	*	*	*	*	*	*	*	*	8	Excellent	[58]
42	Rentsch 2020	*	*	*	*	**	*	*	*	9	Excellent	[59]
43	Rezel-Potts 2021	*	*	*	*	*	*	*	*	8	Excellent	[60]
44	Rodilla 2020	*	*	*	*	**–**	*	*	*	7	Fair	[62]
45	Rosenthal 2020	*	*	*	*	*	*	*	*	8	Excellent	[63]
46	Sardu 2020	*	*	*	*	*	*	*	*	8	Excellent	[64]
47	Selçuk 2020	*	*	*	*	*	*	*	*	8	Excellent	[65]
48	Shah 2020	*	*	*	*	*	*	*	*	8	Excellent	[66]
49	Soleimani 2020	*	*	*	*	**–**	*	*	*	7	Fair	[67]
50	Son 2020	*	*	*	*	*	*	*	*	8	Excellent	[68]
51	Xu 2020	*	*	*	*	*	*	*	*	8	Excellent	[71]
52	Yang 2020	*	*	*	*	**–**	*	*	*	7	Fair	[72]
53	Yuan 2020	*	*	*	*	*	*	*	*	8	Excellent	[73]
54	Zhang 2020	*	*	*	*	**	*	*	*	9	Excellent	[75]
55	Zhong 2020	*	*	*	*	*	*	*	*	8	Excellent	[76]
56	Zhou 2020	*	*	*	*	*	*	*	*	8	Excellent	[77]

S. no.	Reference name	Selection				Compatibility	Outcome		Total score	Quality of the study	References
		Representativeness of the exposed cohort	Selection of the non-exposed cohort	Ascertainment of the exposure	Outcome status at start of study		Assessment of the outcome	Statistical Test (same method for both case and control)			
*Case control*											
1	Bauer 2021	*	*	*	*	**	**	*	9	Excellent	[21]
2	Chen M 2020	*	*	*	*	*	**	**–**	8	Excellent	[29]
3	Choi 2020	*	*	*	*	*	**	*	9	Excellent	[31]
4	Felice 2020	*	**–**	*	*	**–**	**	**–**	5	Fair	[7]
5	Guo 2020	*	**–**	*	*	**–**	**	**–**	5	Fair	[9]
6	Ip 2020	*	*	*	*	**–**	**	**–**	6	Fair	[41]
7	Li 2020	*	*	*	*	**–**	**	**–**	6	Fair	[49]
8	Meng 2020	*	**–**	*	*	**–**	**	**–**	5	Fair	[55]
9	Richardson 2020	*	*	*	*	**–**	**	**–**	6	Fair	[61]
10	Tan 2020	*	*	*	*	**–**	**	**–**	6	Fair	[69]
11	Wang 2020	*	**–**	*	*	**–**	**	**–**	5	Fair	[70]
12	Zeng 2020	*	**–**	*	*	*	**	**–**	6	Fair	[74]

* indicate 01 point and **indicate 02 points regarding quality of particular study

### ACEi/ARB and deaths of COVID-19 patients

**Fig. 2:**
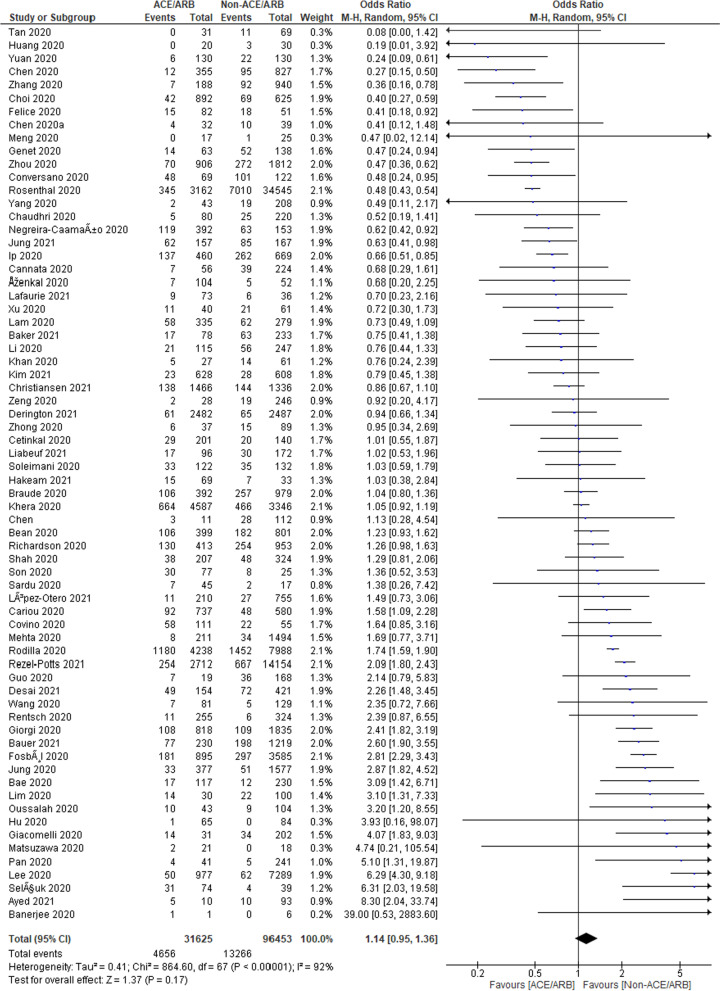
Pooled analysis results using a random effect model ACEi/ARB (forest plot)

A total of 68 studies were included, with a total of 128,078 COVID-19 cases. A total of 31,625 patients were on ACEi/ARB, whereas the remaining 96,453 were in non-ACEi/ARB group. The pooled odds ratio was found to be 1.14 [0.95, 1.36] which indicates a non-signification association of ACEi/ARB in deaths of COVID-19 patients as compared to the non-ACEi/ARB group (Fig. 2). However, the heterogeneity among studies was found to be 92% which is quite high as indicated by *I*^2^ statistics and the Chi-square test (*p* < 0.00001). Therefore, further, sub-group analysis was also done to find out the reasons for heterogeneity.

### Publication bias

**Fig. 3:**
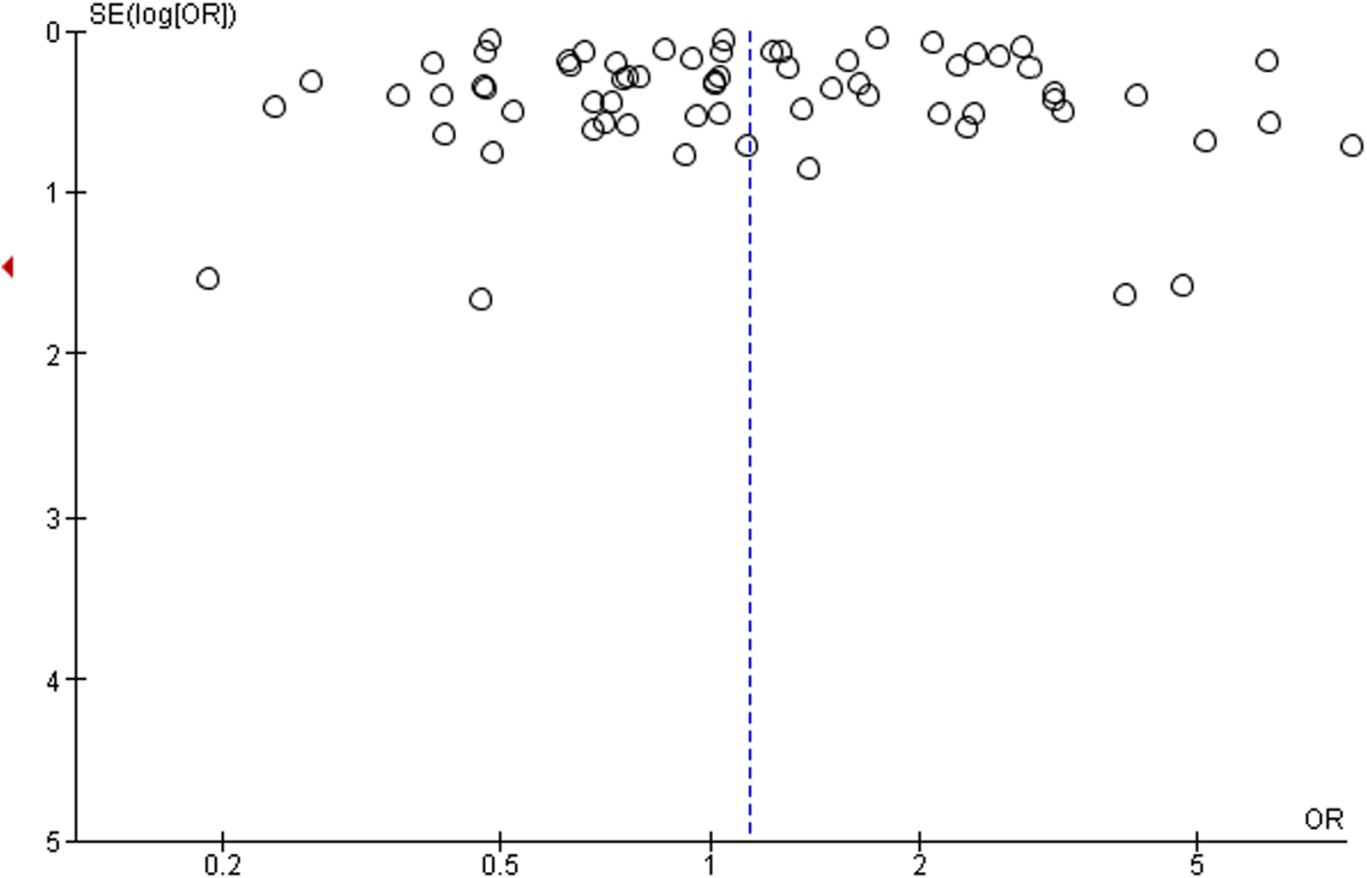
Funnel plot for the assessment of publication bias (ACEi/ARB)

The funnel plot was used to analyze publication bias qualitatively. The shape of the plot revealed some degree of asymmetry (Fig. 3) which indicates the involvement of publication bias.

### Sub-group analysis

The subgroup analysis was done to check the effect of ACEi/ARB individually on the outcome.

#### ACEi

**Fig. 4:**
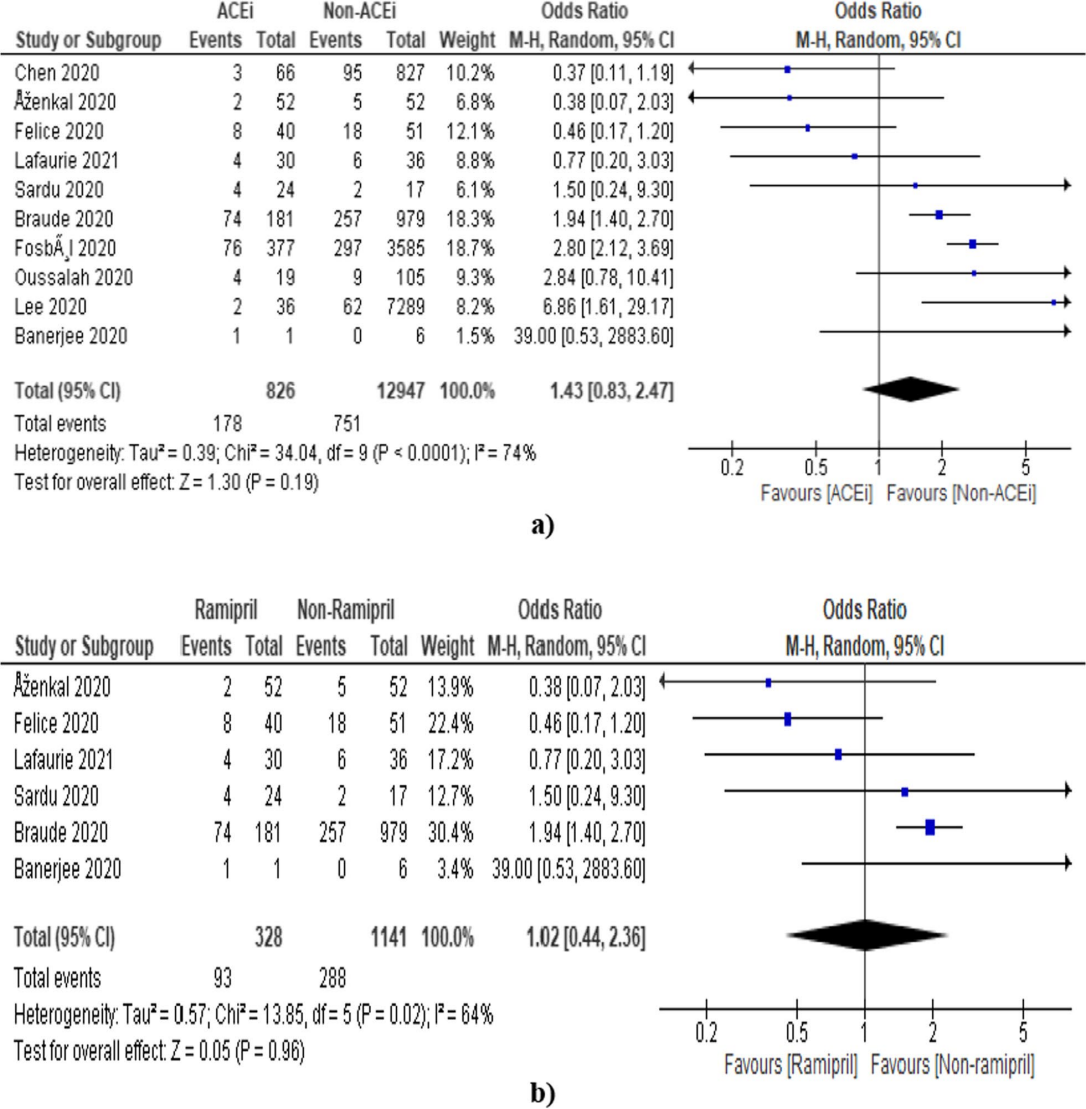
Forest plot showing overall estimate in terms of odds ratio using random effect model **a** ACEi **b** Ramipril

Out of 68 studies, only 10 studies mentioned specifically about ACEi and contain relevant data (Additional file 1: Table S2). The pooled odds ratio was found to be 1.43 [0.83, 2.47] which indicates the non-signification association of ACEi in deaths of COVID-19 patients as compared to the non-ACEi group (Fig. 4a).

**Fig. 5:**
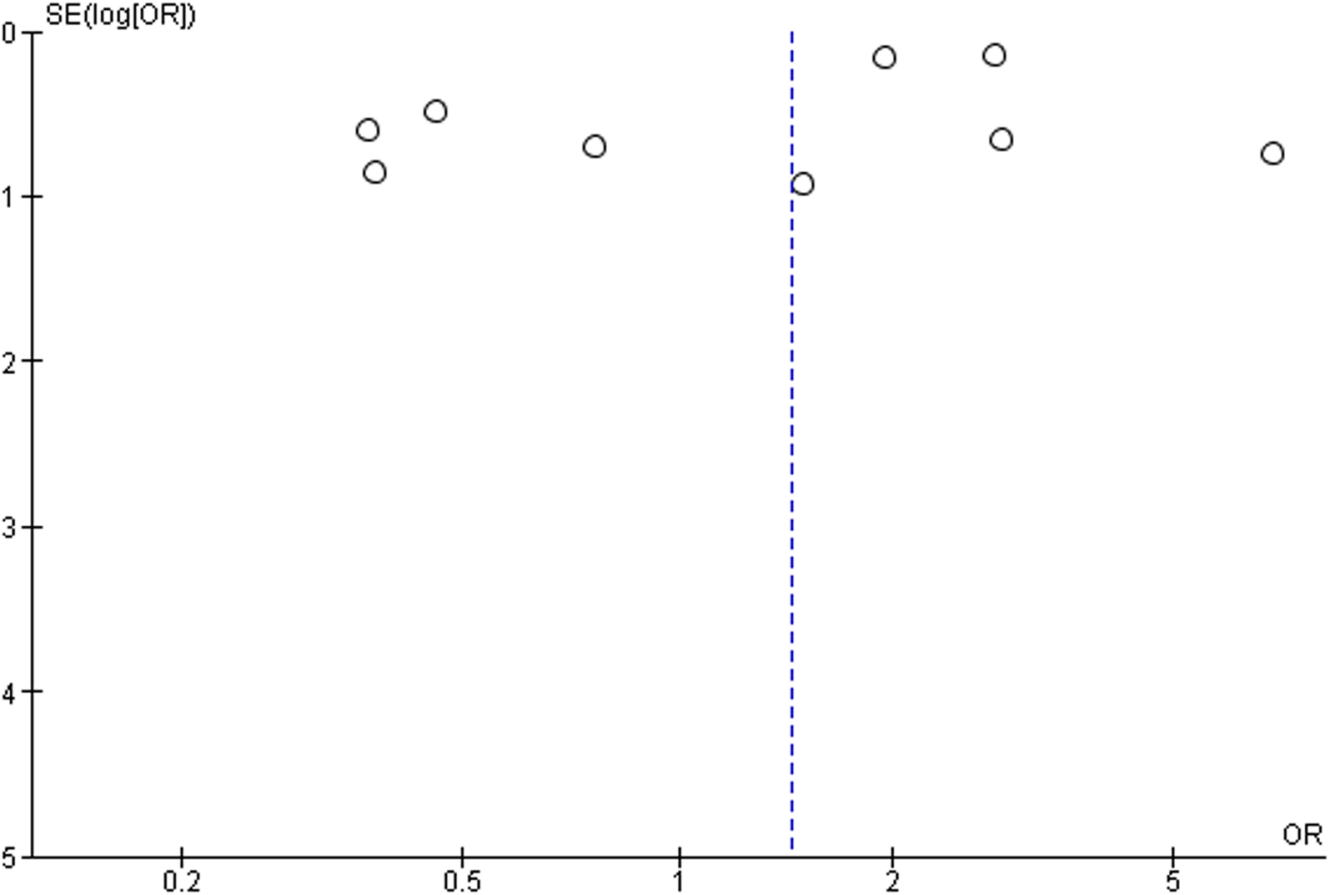
Funnel plot for the assessment of publication bias (ACEi)

Further, we have also tried to check the effect of individual ACEi (captopril, enalapril, lisinopril, perindopril, ramipril, and zofenopril) on the outcome, however, we have got relevant information related to ramipril only (Additional file 1: Table S3). The pooled odds ratio was found to be 1.02 [0.44, 2.36] which indicates the non-significant association of ramipril in deaths of COVID-19 patients as compared to the non-ramipril group (Fig. 4b). The funnel plot also indicated the involvement of publication bias as shown in Fig. 5.

#### ARB

**Fig. 6:**
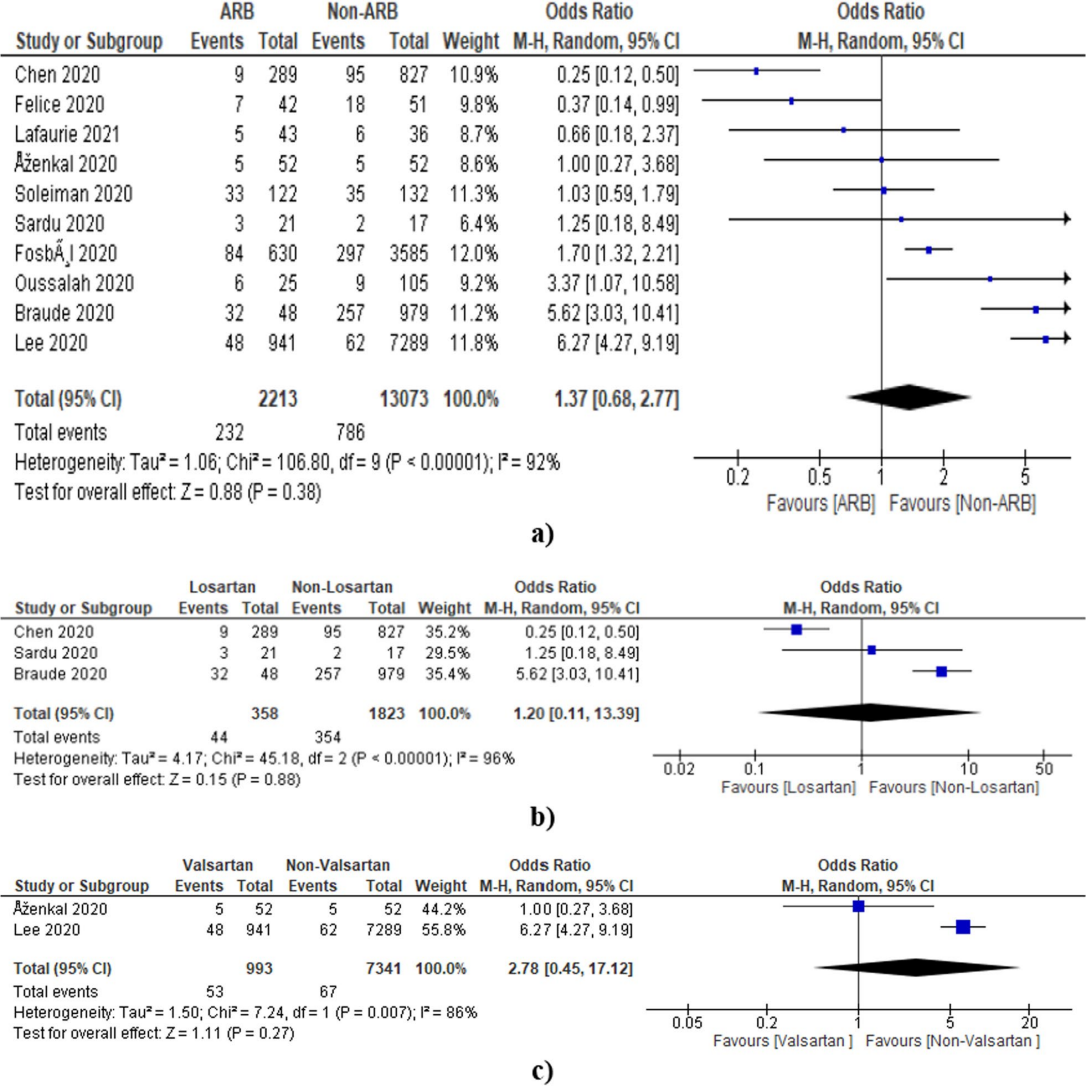
Forest plot showing overall estimate in terms of odds ratio using random effect model **a** ARBs **b** Losartan **c** Valsartan

A total of 10 studies mentioned specifically ARB and contain relevant data (Additional file 1: Table S4). The pooled odds ratio was found to be 1.37 [0.68, 2.77] which indicates the non-significant association of ARB in deaths of COVID-19 patients as compared to the non-ARB group (Fig. 6a). However, the heterogeneity among studies was found to be 92% which is quite high as indicated by *I*^2^ statistics and the Chi-square test (*p* < 0.00001).

**Fig. 7:**
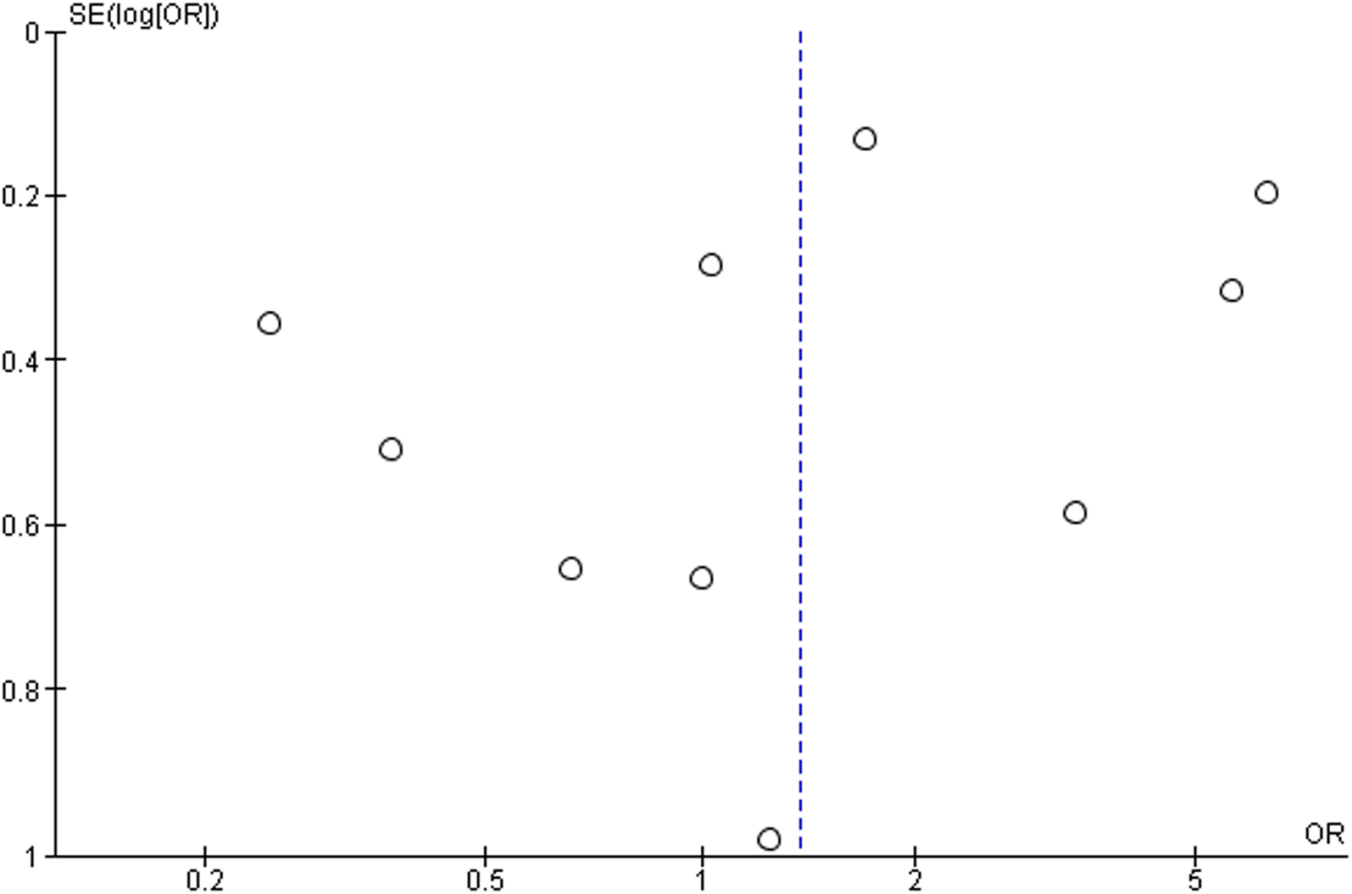
Funnel plot for the assessment of publication bias (ARB)

Further, the effects of individual ARB (candesartan, irbesartan, valsartan, losartan, telmisartan, eprosartan, fimasartan, azilsartan, and olmesartan) on the outcome have also been tried. However, we have found relevant data on losartan (Additional file 1: Table S5) and valsartan only (Additional file 1: Table S6). The pooled odds ratio was found to be 1.20 [0.11, 13.39] and 2.78 [0.45, 17.12] for losartan and valsartan, respectively, which also indicates the non-signification association of losartan and valsartan in the deaths of COVID-19 patients as compared to non-losartan and valsartan group (Fig. 6b, c). The funnel plot indicated involvement of publication bias as shown in Fig. 7.

### Sensitivity analysis

The sensitivity analysis was performed to check the effects of outliers on the outcome.

#### ACEi/ARB

**Fig. 8:**
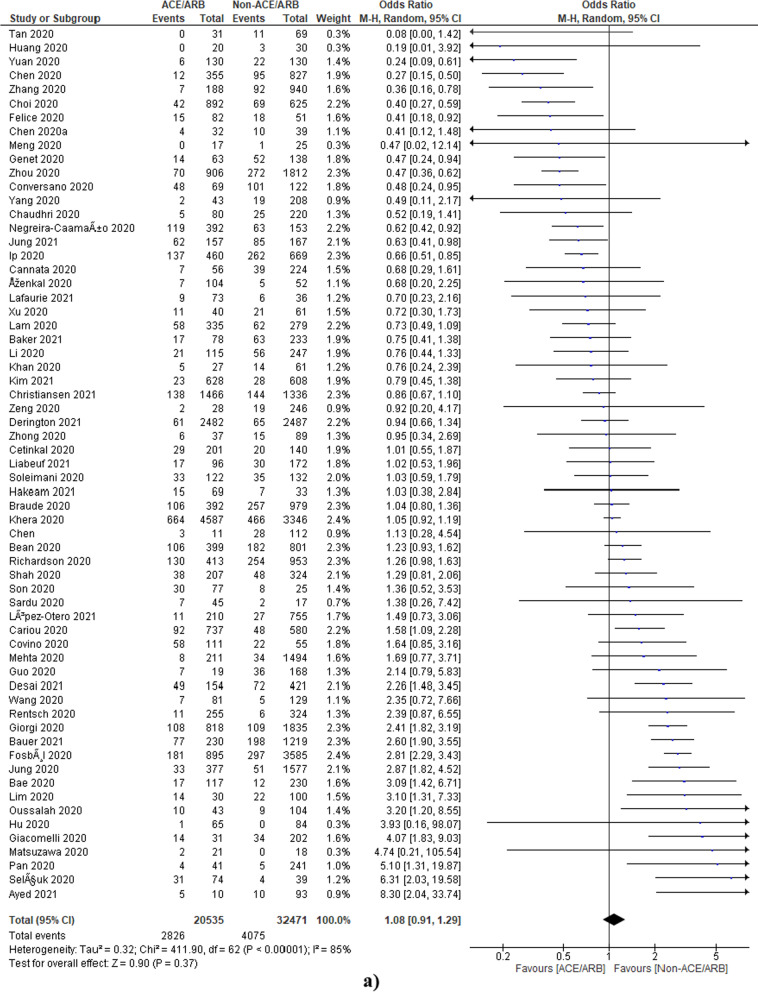

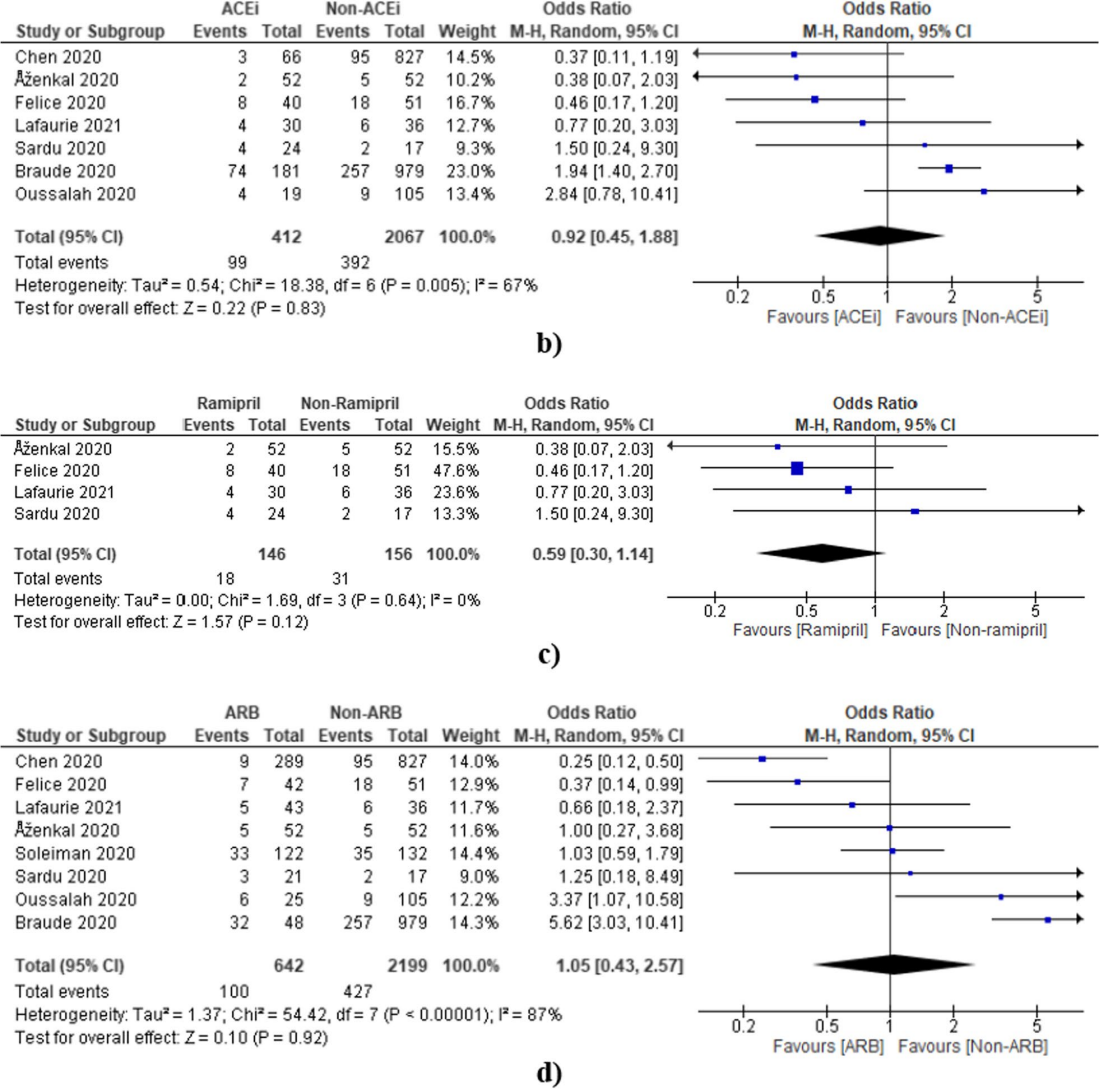
Sensitivity analysis **a** forest plot of ACEi/ARB after exclusion of high sample size studies (Rosenthal 2020 [63], Rodilla 2020 [62], Rezel-Potts 2021 [60], Lee 2020 [48]) and low sample size study (Banerjee 2020 [20]). **b** Forest plot of ACEi after exclusion of high sample size studies (Fosbol 2020, Lee 2020) [8, 48] and low sample size study (Banerjee 2020) [20]. **c** Forest plot of ramipril after exclusion of high sample sizes (Braude 2020) [23] and low sample size (Banerjee 2020) [20]. **d** Forest plot of ARBs after exclusion of high sample size (Fosbol 2020, Lee 2020) [8, 48]

We have identified 4 studies with a high sample size [48, 60, 62, 63] and one study with a low sample size [20]. The analysis was done again after the exclusion of these studies and pooled odds ratios were found to be 1.08 [0.91, 1.29] which also shows non-significant reductions in deaths of COVID-19 patients in the ACEi/ARB group as compared to non-ACEi/ARB group (Fig. 8a).

#### ACEi

The analysis was also done after the exclusion of Fosbol and Lee [8, 48] (high sample size) and Banerjee [20] (low sample size) studies. The pooled odds ratio was found to be 0.92 [0.45, 1.88] which also indicates non-significant reductions in deaths of COVID-19 patients in the ACEi group as compared to the non-ACEi group (Fig. 8b). The overall effect of ramipril was also calculated after the exclusion of Braude [23] (high sample size) and Banerjee [20] (low sample size) studies. The overall odds ratio after exclusion was found to be 0.59 [0.30, 1.14] which also indicates a non-significant reduction in deaths of COVID-19 patients in the ramipril group as compared to the non-rampiril group (Fig. 8c).

#### ARB

The ARB has also shown a non-significant effect after the exclusion of high sample size studies (Fosbol 2020, Lee 2020) [8, 48] (Fig. 8d).

## Discussion

It has been observed that COVID-19 patients with co-morbid conditions such as diabetes (DM), hypertension (HT), or cardiovascular disease (CVD) are more prone to death. There is a number of reported explanations in the literature. The use of medicines could also be one of the reasons. ACEi and ARB are commonly used in hypertensive or cardiovascular disease patients. Both groups of medicines work by inhibiting the RAAS. Angiotensin-converting enzyme inhibitors prevent angiotensin-I from converting to angiotensin-II, whereas angiotensin receptor blockers prevent angiotensin II from acting, resulting in vasodilation and decreased aldosterone output. Angiotensin-converting enzyme-2 (ACE2) is found in a variety of organs, including the alveoli of the lungs, and is related to angiotensin-converting enzyme 1 (ACE1), which plays a role in RAAS [21, 55]. The SARS-CoV-2 uses the angiotensin-converting enzyme (ACE) of the host to enter inside the cell, and some of the classes of drugs (ACEI and ARB) could increase ACE2 expression which can result in increased binding of the SARS-CoV-2. The increased binding of SARS-CoV-2 with the host might result in severe conditions for the patients. Ferrario et al. (2005) have reported safety concerns regarding the use of RAAS inhibitors in COVID-19 patients due to increased ACE2 expression [78]. The COVID-19 hypertensive patients using ACEi/ARB have more tendencies to develop severe pneumonia compared to those not using ACEi/ARB [64]. The literature has shown conflicting findings regarding the use of ACEIs and ARB in COVID-19 patients.

To the best of our knowledge, very few meta-analyses have also been conducted to find out the association of ACEi/ARB in the deaths of COVID-19 patients. Recently, the meta-analysis results of Dai et al. (2021) have reported a non-significant association of ACEIs/ARBs in the deaths of COVID-19 patients. However, data included in this analysis was up to June 20, 2020 [79]. The meta-analysis conducted by Singh et al. (2022) has also shown similar results, however, studies were included up to January 18th, 2021 [80]. Hasan et al. (2020) have conducted a meta-analysis of 24 studies and also reported a non-significant association of ACEIs/ARBs in the deaths of COVID-19 patients [81]. The meta-analysis results of Wang et al. (2021) have concluded that ACEi/ARB treatment was significantly associated with a lower risk of mortality in hypertensive COVID-19 patients. The studies were included up to October 12, 2020 [82]. Recently, the meta-analysis conducted by Azad and Kumar (2022) has shown no significant association of ACEi/ARB in the deaths of COVID-19 patients [83]. We have included 68 observational studies, and the results of our meta-analysis have also shown a non-significant association of ACEi/ARBs in the mortality of COVID-19 patients. We have also tried to find out the effect of individual ACEi/ARBs and also found a non-significant association. Further, the sensitivity analysis results have also shown the non-significant impact of outliers on the outcome.

The failure to publish the results of specific studies due to the direction, nature, or strength of the study findings is known as publication bias. Outcome-reporting bias, time-lag bias, gray-literature bias, full-publication bias, language bias, citation bias, and media-attention bias are all examples of publishing bias in academic articles [84, 85]. The funnel plots of the current investigation have indicated the involvement of publication bias.

Heterogeneity refers to the differences in research outcomes between studies. Heterogeneity is not something to be terrified of; it simply implies that your data are variable. When multiple research projects are brought together for a meta-analysis, it is apparent that differences will be discovered [86]. The current analysis results have also shown heterogeneity among included studies as indicated by *I*^2^ statistics.

## Limitations

We have included seven studies from the medRxiv.org databases that had not yet been peer-reviewed. We saw this as a drawback because peer-reviewers would be able to see more flaws in reporting techniques and other details. The majority of this research, however, was expected to be peer-reviewed. We didn’t find sufficient data to check the effect of all individual ACEi/ARBs on the outcome. The studies published in the English language are only considered. The search is limited to selected databases only. The funnel plots have also indicated the involvement of publication bias.

## Conclusions

In conclusion, to date, the use of ACEi and ARB classes of drugs for the management of co-morbid conditions of COVID-19 patients has not been linked with increased deaths. However, more evidence is required.

## Supplementary Information

**Additional file 1. Table S1:** Search Strategy. **Table S2:** Details of the ACEi therapy (Molecules type). **Table S3:** Details of the ACEi therapy (Molecules type ramipril). **Table S4** Details of the ARB therapy (Molecules type). **Table S5:** Details of the ARB therapy (Molecules type losartan). **Table S6** Details of the ARB therapy (Molecules type valsartan).

## Data Availability

All data are included in Additional file 1.

## Abbreviations

ACEi: Angiotensin-converting enzyme inhibitors
ACE2: Angiotensin-converting enzyme
ARBs: Angiotensin receptor blockers
CVD: Cardiovascular disease
DM: Diabetes mellitus
HT: Hypertension
MeSH: Medical Subject Headings
NOS: Newcastle–Ottawa Scale
PRISMA: Preferred Reporting Items for Systematic reviews and Meta-Analyses
RAAS: Renin–angiotensin–aldosterone system
SARS-CoV-2: Severe acute respiratory syndrome coronavirus 2
2019-nCoV: Novel coronavirus
WHO: World Health Organization
