# Supplementary material for: Drugs acting on the renin–angiotensin–aldosterone system (RAAS) and deaths of COVID-19 patients: a systematic review and meta-analysis of observational studies
Source: Egypt Heart J. 2022 Sep 6;74:64. doi: 10.1186/s43044-022-00303-8 (PMC9448845; doi:10.1186/s43044-022-00303-8)
Supplement: Supplementary file 1 — Additional file 1. Table S1: Search Strategy. Table S2: Details of the ACEi therapy (Molecules type). Table S3: Details of the ACEi therapy (Molecules type ramipril). Table S4 Details of the ARB therapy (Molecules type). Table S5: Details of the ARB therapy (Molecules type losartan). Table S6 Details of the ARB therapy (Molecules type valsartan). [file 43044_2022_303_MOESM1_ESM.docx]

**Drugs acting on the RAAS and Deaths of COVID-19 Patients: A systematic review and Meta-analysis of observational studies**

**Ruchika Sharma^a^, Anoop Kumar^b*^, Jaseela Majeed^c^, Ajit K Thakur^b^ and Geeta Aggarwal^d^*,**

^a^ Centre for Precision Medicine and Pharmacy, Delhi Pharmaceutical Sciences and Research University, New Delhi, 110017, India

^b^ Department of Pharmacology, Delhi Pharmaceutical Sciences and Research University, New Delhi, 110017, India

^c^ Department of Pharmaceutical Management, Delhi Pharmaceutical Sciences and Research University, New Delhi, 110017, India

^d^ Department of Pharmaceutics, Delhi Pharmaceutical Sciences and Research University, New Delhi, 110017, India

**Table S1:** Search Strategy

| The MeSH phrases or keywords that include: “(COVID19) OR (COVID-19) OR (COVID-19 VIRUS INFECTION) OR (COVID19 INFECTION) OR (SARS COVID 19 INFECTION) OR (2019 NOVEL CORONAVIRUS INFECTION) OR (SARS COVID DISEASE) OR (COVID-19 DISEASE) AND (ACE) OR (ARB) OR (ANGIOTENSIN CONVERTING ENZYME) OR (ANGIOTENSIN RECEPTOR BLOCKERS)”. |
| --- |

**Table S2** Details of the ACEi therapy (Molecules type)

| **S no** | **Name of author and year** | **Pril Type** | **Control** | **Death** | **ACEi** | **Death** |
| --- | --- | --- | --- | --- | --- | --- |
| 1 | Banerjee 2020 | Ramipril | 6 | 0 | 1 | 1 |
| 2 | Braude 2020 | Ramipril | 979 | 257 | 181 | 74 |
| 3 | Chen C 2020 | NA | 827 | 95 | 66 | 3 |
| 4 | Felice 2020 | Ramipril | 51 | 18 | 40 | 8 |
| 5 | Fosbol 2020 | Sacubitril | 3585 | 297 | 377 | 76 |
| 6 | Lafaurie 2021 | Ramipril | 36 | 6 | 30 | 4 |
| 7 | Lee 2020 | Captopril, Enalapril, Lisinopril, Perindopril, Ramipril, Zofenopril | 7289 | 62 | 36 | 2 |
| 8 | Oussalah 2020 | Lisinopril, Ramipril, Perindopril, Enalapril | 105 | 9 | 19 | 4 |
| 9 | Sardu 2020 | Ramipril, Enalapril | 17 | 2 | 24 | 4 |
| 10 | Asenkal 2020 | Ramipril, Perindopril | 52 | 5 | 52 | 2 |

**Table S3** Details of the ACEi therapy (Molecules type ramipril)

| **S no** | **Name of author and year** | **Pril Type** | **Control** | **Deaths** | **Ramipril** | **Deaths** |
| --- | --- | --- | --- | --- | --- | --- |
| 1 | Banerjee 2020 | Ramipril | 6 | 0 | 1 | 1 |
| 2 | Braude 2020 | Ramipril | 979 | 257 | 181 | 74 |
| 3 | Felice 2020 | Ramipril, | 51 | 18 | 40 | 8 |
| 4 | Lafaurie 2021 | Ramipril | 36 | 6 | 30 | 4 |
| 5 | Sardu 2020 | Ramipril, Enalapril | 17 | 2 | 24 | 4 |
| 6 | Asenkal 2020 | Ramipril, Perindopril | 52 | 5 | 52 | 2 |

**Table S4** Details of the ARB therapy (Molecules type)

| **S No** | **Name of author and year** | **Sarton Name** | **Control** | **ARB** | **Test** | **Deaths** |
| --- | --- | --- | --- | --- | --- | --- |
| 1 | Braude 2020 | Losartan | 979 | 257 | 48 | 32 |
| 2 | Chen C 2020 | Losartan | 827 | 95 | 289 | 9 |
| 3 | Felice 2020 | Olmesartan | 51 | 18 | 42 | 7 |
| 4 | Fosbol 2020 | Valsartan | 3585 | 297 | 630 | 84 |
| 5 | Hakeam 2021 | Losartan,  Valsartan, Irbesartan, Telmisartan , Candesartan | 33 | 7 | 155 | 1 |
| 6 | Lafaurie 2021 | Irbesartan | 36 | 6 | 43 | 5 |
| 7 | Lee 2020 | Candesartan, Irbesartan, Valsartan, Losartan, Telmisartan, Eprosartan, Fimasartan, Azilsartan, Olmesartan | 7289 | 62 | 941 | 48 |
| 8 | Oussalah 2020 | Candesartan, Telmisartan, Irbesartan,  Losartan,  Valsartan | 104 | 9 | 25 | 6 |
| 9 | Sardu 2020 | Telmisartan, Losartan | 17 | 2 | 21 | 3 |
| 10 | Asenkal 2020 | Valsartan, Kandesartan, Olmesartan | 52 | 5 | 52 | 5 |

**Table S5** Details of the ARB therapy (Molecules type losartan)

| **S No** | **Name of author and year** | **Control** | **Deaths** | **Losartan** | **Deaths** |
| --- | --- | --- | --- | --- | --- |
| 1 | Braude 2020 | 979 | 257 | 48 | 32 |
| 2 | Chen C 2020 | 827 | 95 | 289 | 9 |
| 3 | Sardu 2020 | 17 | 2 | 21 | 3 |

**Table S6** Details of the ARB therapy (Molecules type valsartan)

| **S No** | **Name of author and year** | **Control** | **Deaths** | **Valsartan** | **Deaths** |
| --- | --- | --- | --- | --- | --- |
| 1 | Lee 2020 | 7289 | 62 | 941 | 48 |
| 2 | Soleiman 2020 | 132 | 35 | 122 | 33 |
| 3 | Asenkal 2020 | 52 | 5 | 52 | 5 |
